# A cautious note advocating the use of ensembles of models and driving data in modeling of regional ozone burdens

**DOI:** 10.1007/s11869-024-01516-3

**Published:** 2024-02-05

**Authors:** Jan Karlický, Harald E. Rieder, Peter Huszár, Jan Peiker, Timofei Sukhodolov

**Affiliations:** 1 Department of Atmospheric Physics, Faculty of Mathematics and Physics, Charles University, V Holešovičkách 2, Prague, 180 00 Czech Republic; 2 Institute of Meteorology and Climatology, Department of Water, Atmosphere and Environment, University of Natural Resources and Life Sciences, Gregor-Mendel-Straße 33, Vienna, 1180 Austria; 3 Physikalisch-Meteorologisches Observatorium Davos and World Radiation Center, Dorfstrasse 33, Davos, CH-7260 Switzerland

**Keywords:** Ozone, Chemistry-transport modeling, Air quality, Tropospheric chemistry

## Abstract

We investigate the performance of two widely used chemistry-transport models (CTMs) with different chemical mechanisms in reproducing the ambient maximum daily 8-h average ozone (MDA8 O_3_) burden over Central Europe. We explore a base case setup with boundary conditions (BC) for meteorology from the ERA-Interim reanalysis and chemical BC from CAM-Chem as well as effects of alterations in these BC based on global model fields. Our results show that changes in meteorological BC strongly affect the correlation with observations but only marginally affect the model biases, while changes in chemical BC increase model biases while correlation patterns remain largely unchanged. Furthermore, our study highlights that CTM choice (and choice of chemical mechanism) has a similar or even larger impact on MDA8 O_3_ levels as the impact of altered BC. In summary, our study calls for a multi-model strategy combining different CTM and BC combinations to explore the bandwidth of MDA8 O_3_ distributions and thus uncertainty in hindcasts and future projections, in analogy to climate studies considering ensemble simulations under the same anthropogenic emissions but with slightly different initial conditions.

**Supplementary Information:**

The online version contains supplementary material available at 10.1007/s11869-024-01516-3.

## Introduction

Tropospheric ozone is one of the most important criteria pollutants in the atmosphere, formed through chemical reactions involving nitrogen oxides (NOx), volatile organic compounds (VOC) and solar radiation (Seinfeld & Pandis, 1998). Besides these key components ozone abundances are also strongly modulated by water vapor content and ambient temperature (Doherty et al., 2017), which are highly correlated (Racherla & Adams, 2008), as well as atmospheric mixing (Revell et al., 2015; Huszar et al., 2020b). Today it is well understood that ozone in near-surface air is associated with several negative health impacts, as short-term and long-term exposure is connected with respiratory, cardio-respiratory and cardiovascular mortality (Doherty et al., 2017).

In the recent past, policy efforts have led to a reduction in the emission of anthropogenic ozone precursors, particularly in Europe and the USA, and thus reduced surface ozone burdens (Revell et al., 2015; Chang et al., 2017; Archibald et al., 2020; Mayer et al., 2022). Given the importance of ozone for public health, the future evolution of ozone burdens in light of climate warming and changes in anthropogenic precursor emissions is a topic of active research. The future evolution of the ozone burden is still uncertain (Doherty et al., 2017; Archibald et al., 2020), given that the expected reductions in the emission of anthropogenic ozone precursors (particularly NOx) could be compensated or overcome by a warmer and moister climate (the so-called climate penalty; Colette et al. 2015). To assess future changes in the surface ozone burden in light of different developments of anthropogenic emissions, model simulations performed with chemistry-climate (CCMs) or chemistry-transport models (CTMs) are needed.

Various studies have shown that outputs of CCMs (Colette et al., 2012; Rieder et al., 2015; Revell et al., 2018; Young et al., 2018; Lin et al., 2020) and CTMs (Mar et al., 2016; Karlický et al., 2017; Hogrefe et al., 2018; Im et al., 2018; Otero et al., 2018) are frequently biased compared to observations, leading to additional uncertainties in future projections. The bias in model simulations can stem from a variety of different sources or combinations thereof such as (i) model formulation and incomplete chemical mechanisms, (ii) emission fields, or (iii) meteorological or chemical boundary conditions (BC). While for hindcast simulations “realistic” emission fields and BC exist (from observations and reanalyses, respectively), future simulations rely on free-running global models, leading to additional uncertainties from potential biases in model fields as well as uncertainties emerging from future emission baskets.

To assess the importance of individual contributions to biases between model outputs and observations (and thus uncertainties), targeted sensitivity simulations are needed. Here we expand upon previous work addressing various sources of biases in CTM simulations (Hogrefe et al., 2018; Im et al., 2018; Otero et al., 2018), by addressing systematically the impact of the choice of (i) CTM, (ii) meteorological BC, and (iii) chemical BC on surface ozone burdens in 10-year time slice simulations for the central European domain. Our results motivate a proposal for an ensemble strategy for hindcast simulations and future projections, in analogy to strategies applied in climate modeling, to adequately cover the intrinsic uncertainty space of the underlying modeling chain.

## Methods and data

As our study investigates the influence of the choice of CTM as well as meteorological and chemical BC, we investigate an ensemble of CTM simulations varying these parameters in isolation. We apply two widely used CTMs in this study: (1) the Weather Research Forecasting model with on-line coupling to chemistry (WRF-Chem; Grell et al. 2005), in version 4.0.3, to simulate both meteorological and chemical fields; (2) the Comprehensive Air Quality Model with Extensions (CAMx, v. 6.50; ENVIRON 2018) to simulate chemical fields, driven with meteorological fields taken from the WRF-Chem model.

**Fig. 1:**
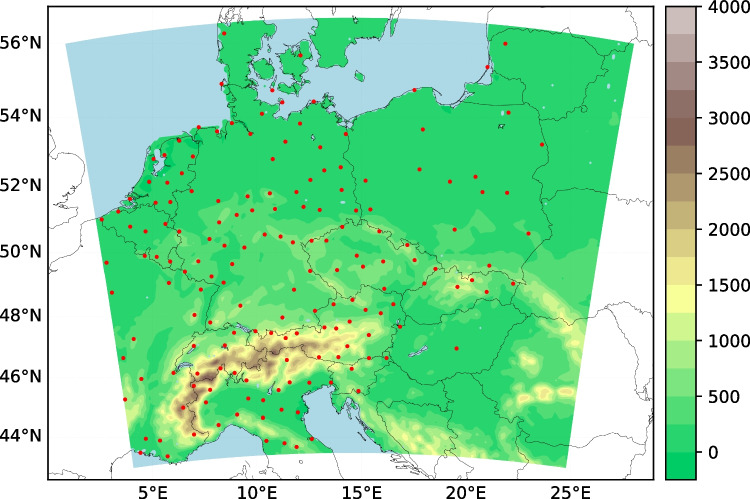
Model domain for WRF-Chem and CAMx simulations with topography [in m a.s.l.] in color coding. Red dots mark observational sites (rural background) from the EEA database used for model evaluation

Simulations for both CTMs were performed for a central European domain (Fig. 1), including 190 $\times$ 166 grid boxes in horizontal direction, with a horizontal grid resolution of 9 km. In vertical dimension, 40 layers are used within the WRF-Chem model, up to the model top at 50 hPa. CAMx simulations were performed with 18 vertical levels up to approximately 11 km, with the 12 lowermost levels identical to those of the WRF-Chem model. The simulation period covers the decade between the years 2007 and 2016.

Below we detail the configuration of the WRF-Chem and CAMx models for this study. For the meteorological components of the WRF-Chem model we have chosen the following parameterizations: the RRTMG scheme (Iacono et al., 2008) for radiation processes; the Purdue Lin scheme (Chen & Sun, 2002) for microphysical processes, and the Grell-3D scheme (Grell, 1993) for convection. Surface layer processes have been described as in the Eta model (Janjić 1994), with land surface exchange parameterized by the Noah land surface model (Chen & Dudhia, 2001), while boundary layer processes have been resolved by the BouLac planetary boundary layer scheme (Bougeault & Lacarrere, 1989). Static land use data were derived from CORINE Land Cover data, version CLC 2012. In grid boxes, where urban land use is dominant, a single-layer urban canopy model (SLUCM; Kusaka et al. 2001) was used with the same urban parameters as stated in Karlické et al. (2018). The choice of physical parameterizations has been based on our experience regarding model performance from recent work (Karlické et al., 2020). For the chemical setup of WRF-chem we have chosen the Regional Acid Deposition Model v.2 (RADM2; Stockwell et al. 1990) for the description of gas-phase chemistry, together with the Modal Aerosol Dynamics Model for Europe and Secondary Organic Aerosol Model (MADE/SORGAM; Schell et al. 2001) for aerosols, with large-scale wet scavenging. Further, photolysis reactions have been parameterized by the Madronich TUV scheme (Madronich, 1987) and NO_x_ emission from lightning were emitted following (Ott et al., 2010).

As CAMx is a standalone model, driven with WRF-Chem meteorological fields, we detail only the chemical setup below. We have adopted the Carbon Bond 5 scheme (CB5; Yarwood et al. 2005) as a gas-phase chemical mechanism, while particulate matter has been described by a static two-mode approach comprising secondary inorganic aerosols by the ISORROPIA model (Nenes et al., 1998), secondary organic aerosols by the SOAP scheme (Strader et al., 1999) and deposition by Zhang et al. (2003, dry) and Seinfeld and Pandis (1998, wet).

Anthropogenic emissions were adapted from the TNO MACC-III database for year 2011 (the central year of our study). We used the Flexible Universal Processor for Modeling Emissions (FUME; Benešová et al. 2018) to transform the annual emission data into model-ready hourly gridded emission files. In WRF-Chem, biogenic emissions were on-line computed by the MEGAN model (Guenther et al., 2006), with the incoming monthly means of temperature and solar radiation updated based on the driving meteorological fields (reanalysis or model, see below). For CAMx, MEGAN was applied offline driven by WRF meteorological data and the resulting biogenic emissions fields were merged with anthropogenic emission fluxes. Note, no dust, sea or biomass burning emissions were considered in our set of simulations, which can account for some model biases compared to observations during large dust or biomass burning episodes given their influence on both chemistry and radiation (e.g. Kong et al. 2015; Liu et al. 2020; Bourgeois et al. 2021).

We used two sets of both meteorological and chemical BC in our simulations with WRF-Chem (hereinafter abbreviated as W) and CAMx (hereinafter abbreviated as C). As a meteorological BC, we used data from the ERA-interim reanalysis (Dee et al., 2011) and bias-corrected outputs of the National Center for Atmospheric Research (NCAR) Community Earth System Model (CESM; Monaghan et al. 2014). For chemical BC, we used CAM-chem global model data (Buchholz et al., 2019), driven by the MERRA2 reanalysis, thus following real weather conditions, and outputs of the free-running SOCOL v.4 Earth System Model (Sukhodolov et al., 2021). The experimental protocol comprises model experiments in a basic setup combining two reanalysis-based BC (ERA-interim and CAM-chem), and simulations with altered meteorological BC (CESM instead of Era-Interim) and altered chemical BC (SOCOL v.4 instead of CAM-chem), as noted in Table 1.

**Table 1:** Setup of individual model simulations analyzed in this study

Experiment	CTM	BC setup	Meteorological BC	Chemical BC
W-E-Cam	WRF-Chem	E-Cam	ERA-Interim	CAM-chem
C-E-Cam	CAMx	E-Cam	ERA-Interim	CAM-chem
W-CESM-Cam	WRF-Chem	CESM-Cam	CESM	CAM-chem
C-CESM-Cam	CAMx	CESM-Cam	CESM	CAM-chem
W-E-Soc4	WRF-Chem	E-Soc4	ERA-Interim	SOCOL v.4
C-E-Soc4	CAMx	E-Soc4	ERA-Interim	SOCOL v.4

**Fig. 2:**
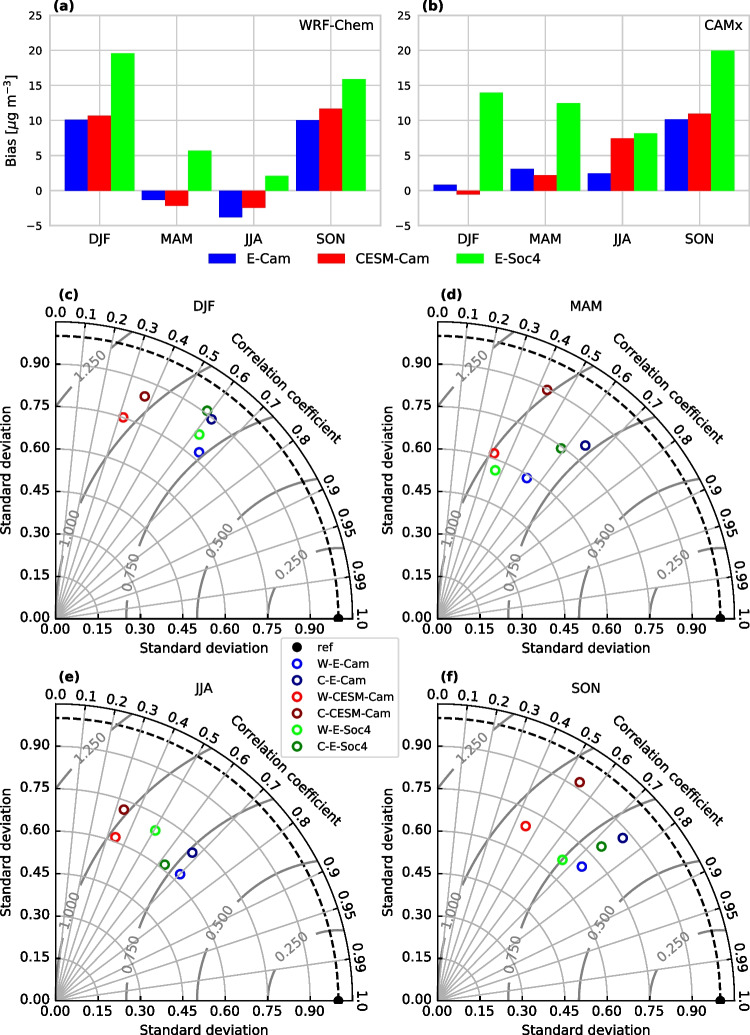
Evaluation of model performance in different configurations (E-Cam, CESM-Cam, E-Soc4) against MDA8 O_3_ observations: Seasonal mean bias for (a) WRF-Chem (W) and (b) CAMx (C) simulations; Seasonal Taylor diagrams for both models combined (c)–(f)

For the evaluation of modeled ozone levels, we use reference data taken from the database of the European Environmental Agency (EEA), which offers data in two data streams, covering different time periods: AirBase (https://www.eea.europa.eu/data-and-maps/data/aqereporting-8, last access: 20 April 2022) and E1a (https://discomap.eea.europa.eu/map/fme/AirQualityExport.htm, last access: 20 April 2022). The selection of stations has been based on data availability over the simulation period and spatial homogeneity. This selection process involved several steps: First, only rural background stations, included in both streams, have been selected which had at least 50% of valid observational records. From this set a few stations were omitted because of suspiciously low values (< 2.5 times the standard deviation of the seasonal mean), others to achieve better spatial homogeneity (excluding iterative stations by determining the distance to the two closest nearby neighbors). The resulting selected stations (#165) are illustrated in Fig. 1.

**Fig. 3:**
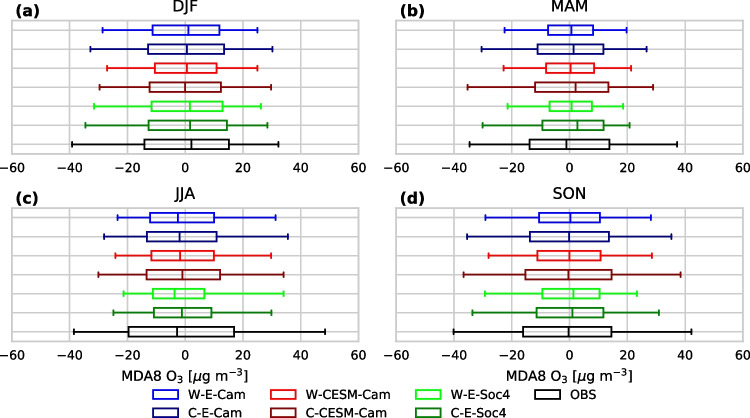
Normalized boxplots (through subtraction of the seasonal mean from individual MDA8 O_3_ values) of the MDA8 O_3_ distribution [$\mu$g m^-3^] for observations and WRF-Chem (W) and CAMx (C) simulations on seasonal basis

## Results

Having detailed our model setup and simulation strategy we turn to the evaluation of CTM performance for 2007–2016 and the influence of changes in model boundary conditions. Figure 2 shows a broad evaluation of model performance with respect to (1) mean bias against observations, (2) correlation between model output and observations and (3) differences in variability between model fields and observations. First, we turn the focus to mean model biases in maximum daily 8-h average ozone (MDA8 O_3_) illustrated in Fig. 2a, b for the base simulation and all sensitivity setups on seasonal basis (DJF, MAM, JJA, SON) for both models. For the base case simulations (E-Cam), biases differ substantially between WRF-Chem and CAMx in all seasons except fall, with generally smaller mean biases in MDA8 O_3_ found for CAMx than for WRF-Chem. For both models, the mean bias is smaller during the warm seasons of the year (MAM and JJA), which also show highest ozone abundances. For the base case, the mean bias in MDA8 O_3_ is found to be within ±10 $\mu$g m^-3^. Next, we turn to the sensitivity simulations with changes in meteorological (CESM-Cam) and chemical BC (E-Soc4). For the CESM-Cam simulations, the mean bias in MDA8 O_3_ does not change substantially compared to the base case during SON–MAM. However, during summer season larger differences emerge, with opposite sign for WRF-Chem (reduced bias) and CAMx (enhanced bias). These summertime difference will be explored in greater detail further below. Turning to the E-Soc4 simulations we find in all seasons a substantially enhanced mean bias in MDA8 O_3_ for both WRF-Chem and CAMx (up to 20 $\mu$g m^-3^). Interestingly however, compared to other seasons, JJA still shows the smallest mean bias for both models (less than 10 $\mu$g m^-3^).

**Fig. 4:**
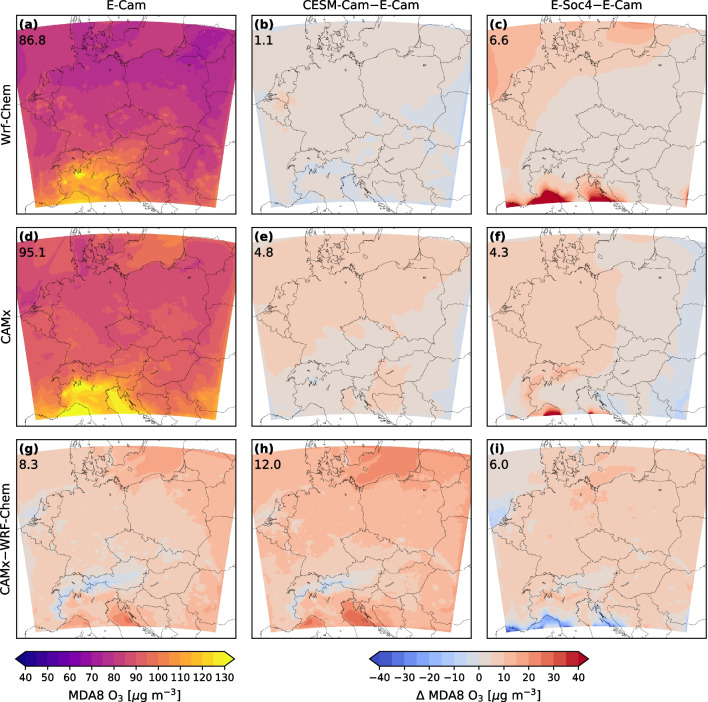
Summertime mean MDA8 O_3_ levels [$\mu$g m^-3^] over the central European domain and differences [$\Delta$] among models and model setups: (a) mean MDA8 O_3_ for WRF-Chem in E-Cam configuration, (b) difference between WRF-Chem in CESM-Cam and E-Cam configuration, (c) as (b) but for differences between E-Soc4 and E-Cam configurations; (d)–(f) as (a)–(c) but for CAMx integrations; (g) difference between WRF-Chem and CAMx in E-Cam configuration, (h) as (g) but for models in CESM-Cam configuration, (i) as (g) but for models in E-Soc4 configuration

Focusing on the mean MDA8 O_3_ bias, these initial results highlight a greater sensitivity to changes in chemical than meteorological BC. To address the robustness of this result we repeat the evaluation for subsets of the observational sites and corresponding model grid cells. To this end we apply a random sampling approach selecting 50 times 50 stations from the total stack and compute the seasonal mean bias across CTM–BC combinations. While the absolute mean bias varies slightly in magnitude among the sub-samples, the identified pattern in seasonal mean MDA8 O_3_ biases emerges as robust feature (see Fig. 5 in the Appendix). Therefore, we continue our analysis considering the originally selected subset of 165 stations.

After having explored the model mean biases we turn the focus to differences in the observation-model correlation and variance. These are explored in the Taylor diagrams in Fig. 2c–f along with the centered root mean square errors (CRMSE). The CRMSE is given by the distance between the reference and model points in the diagram and describes the different phase, structure and amplitude of variations contributing to the root mean square error (Taylor, 2001).

In terms of correlation we find that these are higher for the base simulations, with a correlation coefficient (CC) of about 0.7, than the sensitivity simulations, while among the latter correlation is particularly poor for simulations with altered meteorological BC (CESM-Cam). Across seasons, simulations with the CESM-Cam setup show correlation coefficients that are roughly half those of the base case and E-Soc4 setups (CC mostly 0.3–0.4). In contrast, simulations in E-Soc4 configuration show only slightly reduced correlation compared to the base case simulations, indicating a minor influence of the alteration of chemical BC. Regarding CRMSE we find similar results as for correlation. CRMSE progressively increases from the base case to simulations under E-Soc4 and CESM-Cam highlighting again a dominant role of alterations in meteorological BC. The Taylor diagrams reveal that for all simulations the variability obtained from models is lower than for observed values. Further, we find a larger influence on variability of CTM choice than through the choice of meteorological or chemical BC. Generally CAMx simulations show larger variability in seasonal MDA8 O_3_ (normalized standard deviation between 0.7 and 0.9) than simulations performed with WRF-Chem (0.6–0.8).

To explore this further, we illustrate the width of the seasonal MDA8 O_3_ distributions for observations and model simulations in normalized boxplot illustrations in Fig. 3, which we create by subtracting the seasonal mean from the individual MDA8 O_3_ values. For convenient reference we show the full probability density functions in Fig. 6 of the Appendix. Figure 3 reiterates that model simulations show across seasons a narrower MDA8 O_3_ distribution than observations and that among models the distributions obtained with WRF-Chem are narrower than those obtained with CAMx. Considering that ozone levels vary strongly with weather conditions, and that these are identical for both CTMs in the base case, we conclude that mechanisms for chemical ozone production in CAMx are more sensitive to weather conditions than mechanisms within WRF-Chem. Furthermore, for both models base case simulations and simulations with altered meteorological BC show only minor differences in distribution width, while simulations with altered chemical BC yield substantially narrower distributions, especially during the warm period of the year. This indicates that elevated ozone backgrounds resulting from global model chemical BC are overlaying the ambient MDA8 O_3_ variability driven by meteorology.

Next we present the geographical distribution of the differences between individual model simulations under different BC. Given the importance of summer as prime ozone season, we focus this analysis on JJA and evaluate differences among models and model setups for summertime mean MDA8 O_3_ (Fig. 4) and exceedances of the European target value for the protection of human health, MDA8 O_3_ above 120 $\mu$g m^-3^ (Fig. 7 in the Appendix). In the main body of the text we focus on mean MDA8 O_3_; however, we note that results obtained for exceedance days are in general agreement with findings for the mean state.

We start by contrasting the base case simulations for WRF-Chem (Fig. 4a; domain average MDA8 O_3_, hereinafter DA: 86.8 $\mu$g m^-3^) and CAMx (Fig. 4d; DA: 95.1 $\mu$g m^-3^). Here the differences between the two CTMs are larger in the DA and regionally (Fig. 4g; $\Delta$DA: 8.3 $\mu$g m^-3^) than for individual CTMs between the base case and simulations driven with alternate meteorological BC (Fig. 4b, e; $\Delta$DA: 1.1 and 4.8 $\mu$g m^-3^). This highlights the sensitivity of simulated MDA8 O_3_ on CTM choice. The importance of CTM choice is further underlined by our finding of larger DA and regional differences between WRF-Chem and CAMx when driven with alternate meteorological BC (Fig. 4h; $\Delta$DA: 12.0 $\mu$g m^-3^). This illustrates the increased uncertainty in MDA8 O_3_ projections resulting from the combination of model choice and (global model dependent) climate realization. The effects of alternate chemical BC are on average less CTM dependent (Fig. 4c, f; $\Delta$DA: 6.6 vs. 4.3 $\mu$g m^-3^) but for a given CTM regionally much larger than those of alternate meteorological BC (Fig. 4b, e). Between CTMs however the differences emerging in simulations driven with different chemical BC are (in the DA as well as regionally) smaller (Fig. 4i; $\Delta$DA: 6.0 $\mu$g m^-3^) than in simulations driven with different meteorological BC (Fig. 4h; $\Delta$DA: 12.0 $\mu$g m^-3^).

## Discussion and conclusions

In our study we explore the overall model performance of two widely used CTMs over the central European domain as well as the influence of alterations in meteorological and chemical BC. The evaluation of base case (E-Cam) simulations shows a similar accuracy/bias and similar seasonal differences in correlation between model output and observations as found in previous studies (e.g., Mar et al. 2016; Karlický et al. 2017; Im et al. 2018). While previous studies have frequently explored either the influence of chemistry through BC from global models (Tang et al., 2007; Hogrefe et al., 2018; Andersson et al., 2015; Tang et al., 2021) and/or reductions/enhancements in direct emissions or lateral boundary conditions (e.g., Jiménez et al. 2007; Fast et al. 2014; Andersson et al. 2015; Pendlebury et al. 2018; Im et al. 2018) or the influence of meteorology through alternate BC from climate models (Lacressonnière et al., 2012) our study investigates both the effects of changes in chemical and meteorological BC. Furthermore, our analysis considers 10-year time slices, which is a simulation length substantially longer than in most previous studies exploring uncertainties in CTM fields.

Moving from the base case to simulations with alternate meteorological BC, we find a substantial decrease in correlation with observations but modest change in bias across seasons, which is in broad agreement with results by Lacressonnière et al. (2012). In contrast, moving to alternate chemical BC increases the seasonal bias but does not substantially affect the correlation pattern with observations. The strong effect of chemical BC (or emission changes) on pollutant concentrations is in agreement with results of previous work (Fast et al., 2014; Mar et al., 2016; Hogrefe et al., 2018; Im et al., 2018). While the first effect is caused by a discrepancy between real weather and model weather, the second effect is caused by elevated ozone abundances in the CCM which penetrate to the model domain through chemical BC. These findings reflect also in differences in the width of the MDA8 O_3_ distribution as measure of intrinsic variability and highlight the importance of meteorology as driver of the temporal variability in MDA8 O_3_ and the importance of chemical BC for setting the ozone background.

Contrasting the results obtained between the two CTMs, we find a higher sensitivity of the CAMx model to ambient meteorology. This manifests in a better captured annual cycle of MDA8 O_3_ because of more uniform seasonal biases, larger variability in MDA8 O_3_ and peak MDA8 O_3_ compared to WRF-Chem, and larger inter-model differences emerging in warmer parts of the model domain. These findings are in broad agreement with other recent studies applying these models to study ozone abundances in the European domain (e.g., Huszar et al. 2020a, 2021; Flandorfer et al. 2020) and other studies exploring differences among CTMs or chemical mechanisms (Knote et al., 2015; Gupta & Mohan, 2015; Mar et al., 2016; Sharma et al., 2017; Georgiou et al., 2018; Im et al., 2018).

In summary, our results highlight the different sensitivities of CTMs to changes in meteorological or chemical BC along with the importance of general CTM choice for regional MDA8 O_3_ projections. Our findings illustrate that CTM choice can have in terms of model biases and variability similar (or even larger) effects as alterations of meteorological or chemical BC. This is an important finding as it also applies to studies seeking to explore changes in future regional air quality (leaving alone uncertainties emerging from scenarios for future precursor emissions), where CTMs have to largely rely on their global free-running counterparts. Global CCMs are known to have issues in reproducing tropospheric ozone abundance, variability, trends, and extremes (Revell et al., 2018; Young et al., 2018; Zhang & Cui, 2022), which is also influenced by the lack of spatial coverage of tropospheric observations (Gaudel et al., 2018). Moreover, the inter-model spread of dynamical responses to future climate change will also affect these parameters and further increase the uncertainty. For example, Zhang and Cui (2022) showed that global model simulations constrained by reanalysis data outperform the free-running simulations in terms of inter-annual variability and decadal trends of surface ozone. Our study shows that future projections of the regional air quality trends will, therefore, accumulate all the shortcomings of the parent global models plus the sensitivities of CTM responses to these unavoidably biased dynamical and chemical BC. This calls for a thorough ensemble strategy combining different CTM and BC combinations to explore the bandwidth and thus uncertainty in MDA8 O_3_ projections.

### Supplementary Information

Below is the link to the electronic supplementary material.

Supplementary file 1 (pdf 21 KB)

Supplementary file 2 (pdf 50 KB)

Supplementary file 3 (pdf 2499 KB)

## Data Availability

The source code of the WRF-Chem model is publicly available (after registration) at https://www2.mmm.ucar.edu/wrf/users/download/get_source.html, WRF (2021). CAMx version 6.50 is available at https://www.camx.com/download/, ENVIRON (2018). The complete ozone surface data selected from model outputs used in the study are available at Czech National Repository via https://doi.org/10.48700/datst.ev7ej-gv255, with Creative Commons Attribution 4.0 International License.
