# Supplementary figures and images for: A cautious note advocating the use of ensembles of models and driving data in modeling of regional ozone burdens
Source: Air Qual Atmos Health. 2024 Feb 5;17(7):1415–24. doi: 10.1007/s11869-024-01516-3 (PMC11322370; doi:10.1007/s11869-024-01516-3)

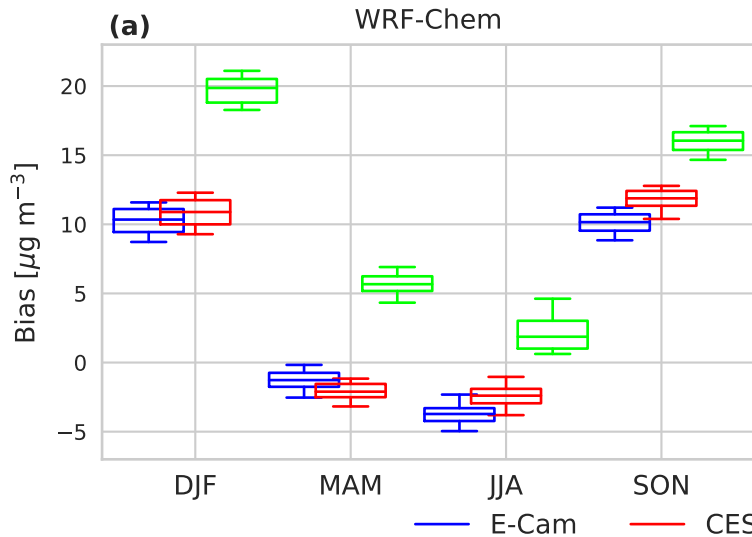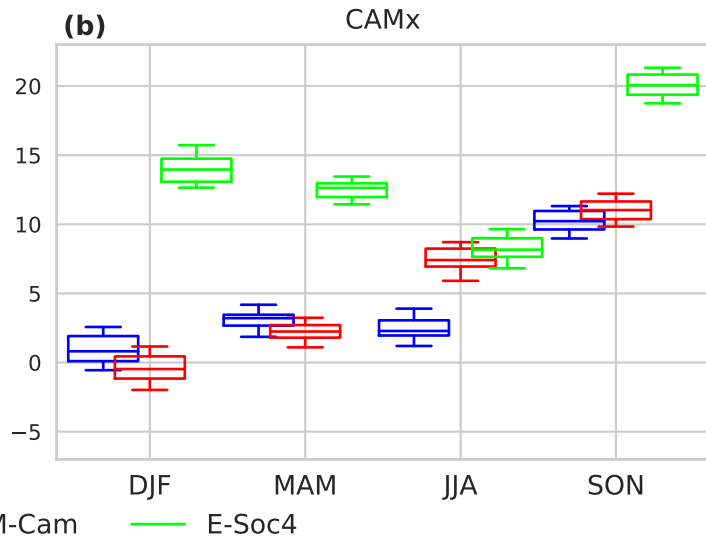

Supplement: Supplementary file 1 — (pdf 21 KB) [file 11869_2024_1516_MOESM1_ESM.pdf]

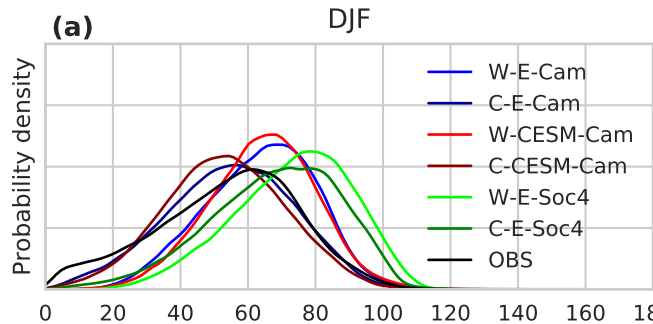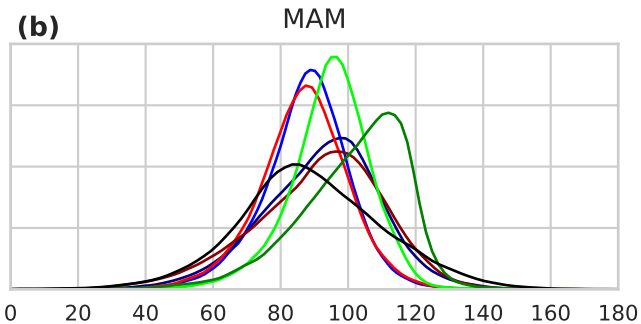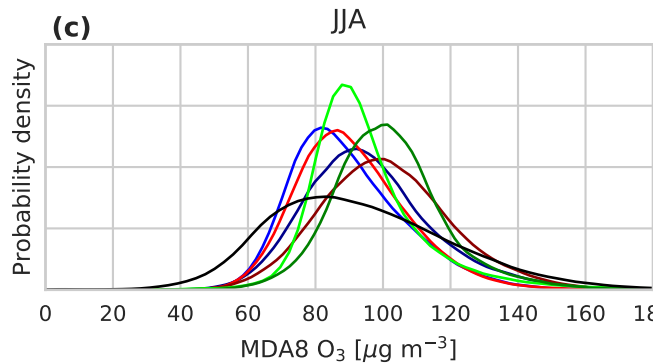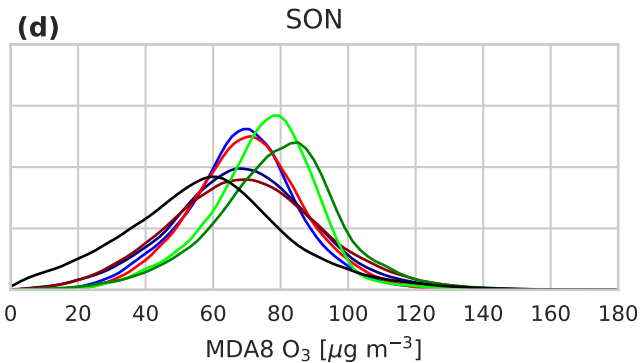

Supplement: Supplementary file 2 — (pdf 50 KB) [file 11869_2024_1516_MOESM2_ESM.pdf]

E-Cam

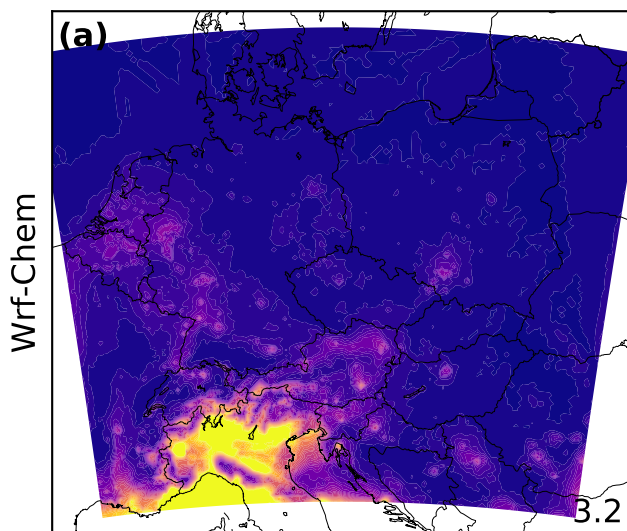

CESM-Cam—E-Cam

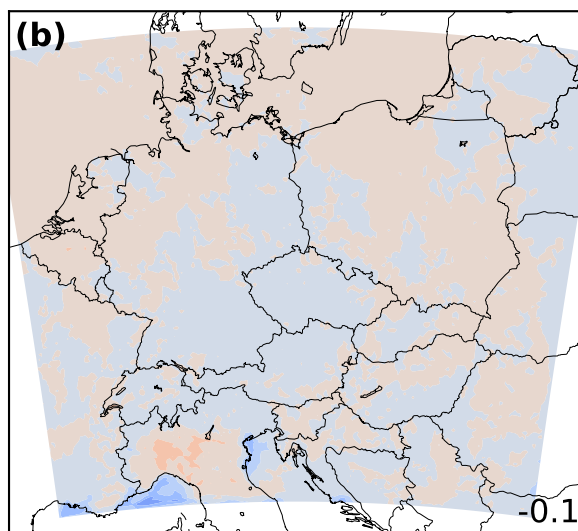

E-Soc4—E-Cam

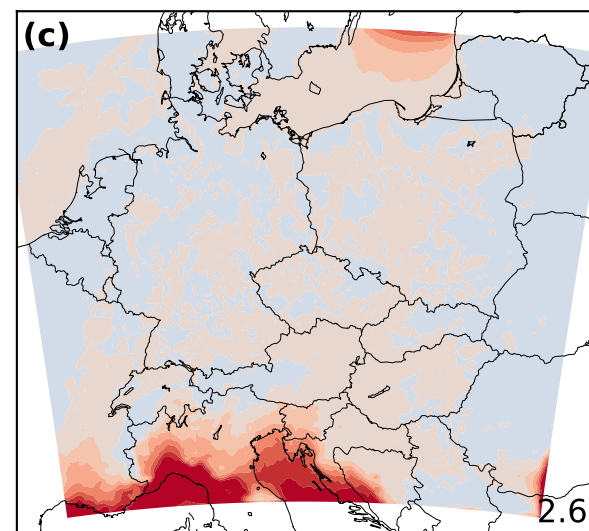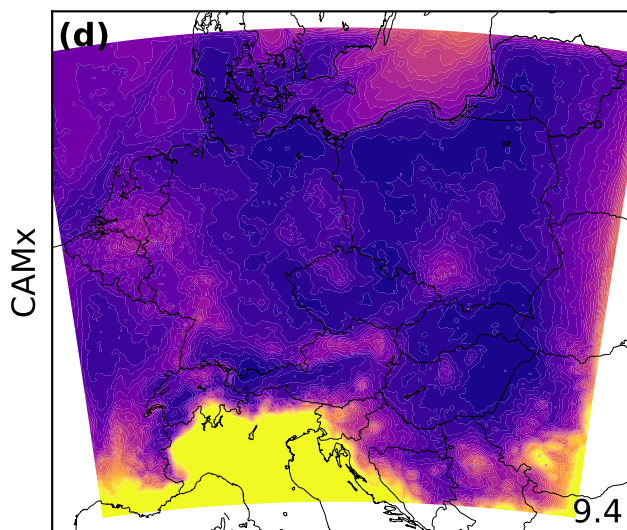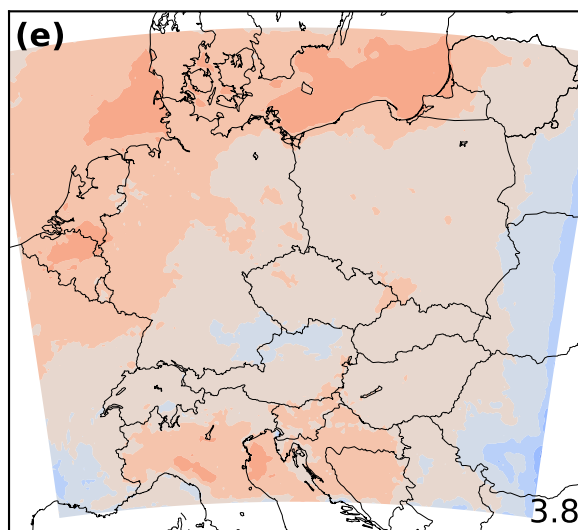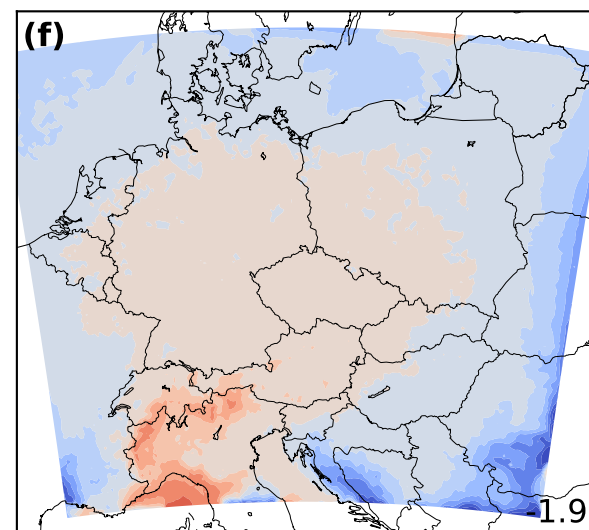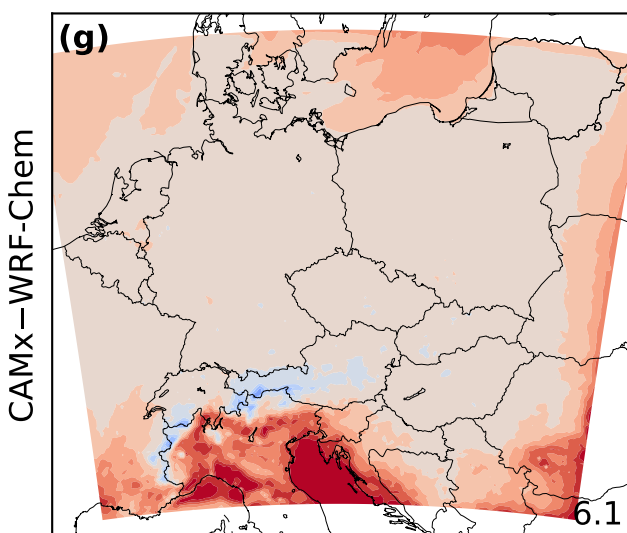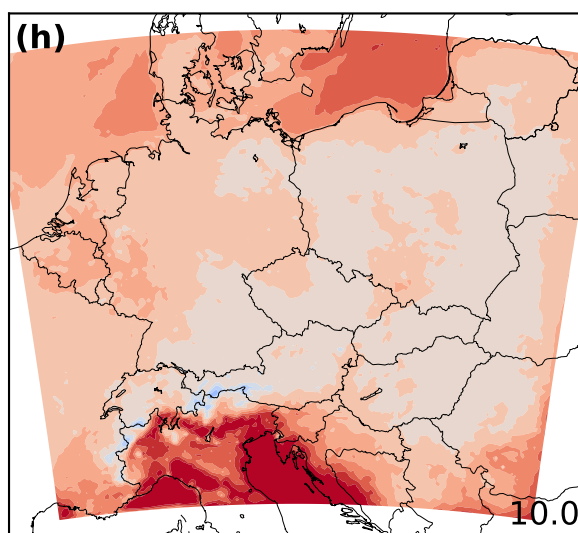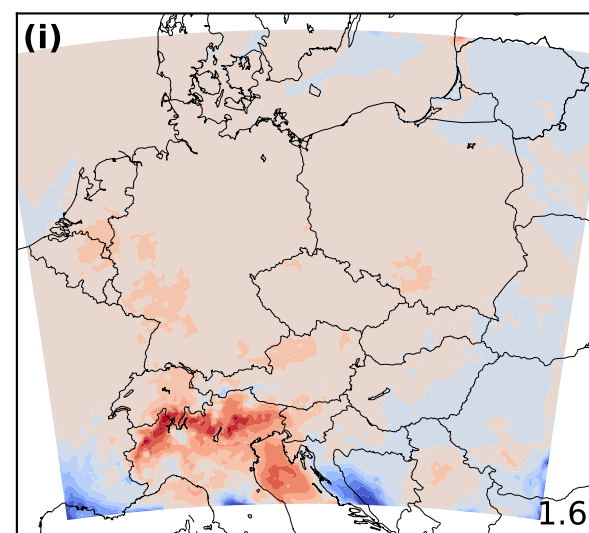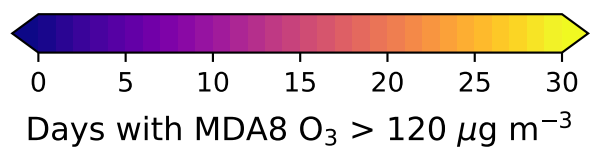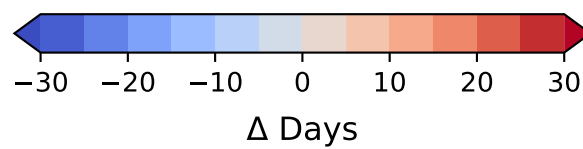

Supplement: Supplementary file 3 — (pdf 2499 KB) [file 11869_2024_1516_MOESM3_ESM.pdf]
